# Replating Induces mTOR-Dependent Rescue of Protein Synthesis in Charcot–Marie–Tooth Diseased Neurons

**DOI:** 10.1523/ENEURO.0337-25.2026

**Published:** 2026-03-27

**Authors:** Julianna Koenig, Alexys McGuire, Yara Homedan, Jessica Alberhasky, Daniel W. Summers

**Affiliations:** ^1^Interdisciplinary Graduate Program in Genetics, University of Iowa, Iowa City, Iowa 52242; ^2^Department of Biology, University of Iowa, Iowa City, Iowa 52242; ^3^Iowa Neuroscience Institute, University of Iowa, Iowa City, Iowa 52242

**Keywords:** axon degeneration, axon regeneration, Charcot–Marie–Tooth disease, neurodegeneration, protein synthesis, tRNA synthetase

## Abstract

Charcot–Marie–Tooth disease (CMT) is an inherited peripheral neuropathy characterized by sensory dysfunction and muscle weakness, manifesting in the most distal limbs first and progressing more proximal. Over a hundred genes are currently linked to CMT with enrichment for activities in myelination, axon transport, and protein synthesis. Mutations in tRNA synthetases cause dominantly inherited forms of CMT, and animal models with CMT-linked mutations in these enzymes display defects in neuronal protein synthesis. Rescuing protein synthesis in CMT-mutant neurons could offer exciting therapeutic options beyond symptom management. To address this need, we expressed CMT-linked variants of tyrosyl-tRNA synthetase (YARS–CMT) in primary mouse sensory neurons derived from both male and female embryos and evaluated impacts on protein synthesis and cell viability. YARS–CMT expression reduced protein synthesis in these neurons prior to the onset of caspase-dependent axon degeneration and cell death. To determine how YARS–CMT expression affects axon outgrowth, we dissociated and replated these neurons to stimulate axon regeneration. To our surprise, axonal regrowth occurred normally in replated YARS–CMT neurons. Moreover, replating YARS–CMT neurons rescued protein synthesis. Inhibiting mammalian target of rapamycin suppressed rescue of protein synthesis after replating, consistent with its significant role in protein synthesis during axon regeneration. These discoveries identify new avenues for augmenting protein synthesis in diseased neurons and restoring protein synthesis in CMT or other neurological disorders.

## Significance Statement

Peripheral neuropathies represent a challenging threat to human health, impacting quality of life for millions with limited treatment options beyond symptom management. Charcot–Marie–Tooth disease (CMT) is the most common inherited peripheral neuropathy with some causative mutations identified in an enzyme family of tRNA synthetases. We use a cellular model of this disease to understand mechanisms underlying CMT and identify novel ways to protect neuron function. We observe severe defects in protein synthesis in our model followed by axon degeneration. Most importantly, we rescue protein synthesis by stimulating a regenerative growth program in these neurons which promotes normal axon elongation despite CMT mutations. Restoring protein synthesis will have broad relevance to many neurological disorders and warrants additional investigation.

## Introduction

Neurons of the peripheral nervous system (PNS) are responsible for relaying somatosensory information to the central nervous system (CNS) and stimulating muscle fibers. Both PNS and CNS neurons are postmitotic and must sustain these vital functions for an organism's entire lifespan. Neurons establish connections throughout the body via long axons, extending more than a meter in the human PNS. This extreme length coupled with a high metabolic demand renders axons especially vulnerable to damage and stress (Coleman and Hoke, 2020). As such, peripheral neuropathies are characterized by progressive and sometimes irreversible damage to the PNS, causing severe disability and diminished quality of life (Hanewinckel et al., 2016). With treatment options focused on symptom management, understanding the mechanistic basis of peripheral neuropathies will identify new opportunities for halting disease progression or even reversing PNS damage.

Charcot–Marie–Tooth disease (CMT) is the most common inherited peripheral neuropathy with an estimated prevalence of 1 in 2,500 to 1 in 10,000 predicted from epidemiological studies (Skre, 1974; Martyn and Hughes, 1997; Barreto et al., 2016). CMT is characterized by progressive distal muscle weakness and sensory loss arising from the gradual dysfunction of peripheral axons through demyelination or intrinsic degeneration. These pathological mechanisms form the basis of CMT categorization into demyelinating (CMT1) or axonal (CMT2) forms, with disorders exhibiting features of both classified as intermediate CMT. Further subcategorization within each form is defined by the causative mutations (Pareyson and Marchesi, 2009).

Aminoacyl-tRNA synthetases (aaRS) represent the largest gene family implicated in CMT, contributing to both CMT2 and dominant forms of intermediate CMT (DI-CMT; Zhang et al., 2021). AaRS covalently link tRNAs with their cognate amino acid, thereby charging tRNAs for polypeptide synthesis at the ribosome. CMT-linked mutations predominantly occur in the aaRS catalytic domain suggesting loss of aminoacylation activity as the underlying cause; however there are notable exceptions and discrepancies reported for most aaRS (Zhang et al., 2021). For example, autosomal dominant mutations in tyrosyl-tRNA synthetase (YARS) cause DI-CMTC, yet there is a disconnect between aminoacylation activity and neuropathy. Of the three most studied CMT-linked variants of YARS, G41R and 153–156delVKQV decrease aminoacylation, while E196K displays normal aminoacylation; however, all three provoke neuropathy in animal models (Jordanova et al., 2006; Storkebaum et al., 2009; Froelich and First, 2011; Hines et al., 2022). Gain-of-function interactions for these CMT variants may also contribute to neuropathy beyond directly impacting protein synthesis (Blocquel et al., 2017; Bervoets et al., 2019; Ermanoska et al., 2023; Rhymes et al., 2024).

Despite variable effects on aminoacylation activity, *Drosophila* models of CMT–YARS report reductions in global translation from motor and sensory neurons expressing any of the abovementioned YARS variants (Niehues et al., 2015) pointing to significant roles for protein homeostasis in aaRS-CMT neuropathies. Yet there remain important gaps in knowledge. For example, protein synthesis is critical for axon outgrowth during neurodevelopment, yet symptom onset in patients with aaRS-CMT variants usually occurs during late childhood or early adolescence when PNS axons have already innervated their targets.

In this study, we identify protein synthesis defects induced by CMT mutations in tyrosyl-tRNA synthetase (YARS) using a primary sensory neuron model. Protein synthesis defects preceded the onset of axon degeneration and coincided with caspase activation. We used a replating procedure that induces axon regeneration to assess how these CMT–YARS variants affect axon regrowth. While chemical inhibition of protein synthesis suppressed axonal regrowth, we were surprised to observe normal axon regrowth in CMT–YARS neurons. Moreover, replating reversed protein synthesis defects in CMT–YARS neurons through mammalian target of rapamycin (mTOR) signaling. We propose that developmental outgrowth pathways triggered during replating protect neurons from CMT–YARS and that these safeguards gradually diminish as PNS neurons mature, leading to disease onset.

## Materials and Methods

### Plasmids and reagents

TimeStamp (TS) was amplified from PSD95-TS-YFP (Addgene plasmid #43335; http://n2taddgene:4225; RRID:Addgene_42225) and subcloned downstream of the human ubiquitin promoter with Gibson cloning. Human tyrosyl-tRNA synthase (YARS) and variants were subcloned downstream of the human ubiquitin promoter with Gibson cloning. Sequences for generating sgRNAs targeting mouse Bax were #1 5′ GTTTCATCCAGGATCGAGCA3′ and #2 5′ TTGCTGATGGCAACTTCAAC 3′. Scramble sgRNA sequences, Cas9 expression plasmid, Bcl-xL, and myristoylated mScarlet were used as previously described (Danos et al., 2025). Antibodies for Western immunoblotting were used as follows: anti-YARS (Bethyl Laboratories; RRID: AB_2631459; 1:1,000), anti-Tuj1 (BioLegend; RRID: AB_2562570; 1:10,000), anti-GAPDH (Santa Cruz Biotechnology; RRID: AB_10847862; 1:500), anti-phospho-S6 ribosomal protein (Ser235/236; Cell Signaling Technology; RRID: AB_331679 1:1,000), anti-S6 ribosomal protein (Cell Signaling Technology; RRID: AB_2238583 1:500), and anti-GFP (Thermo Fisher Scientific; RRID: AB_221569). Revert 700 total protein stain and wash is from LI-CORbio. Chemicals were from the following vendors. Torin 1, danoprevir, Actinomycin D, 8-CPT-Cyclic AMP, and anisomycin were from Cayman Chemical. Chemicals were prepared as a stock solution and stored as aliquots per recommendations from the vendor. Each aliquot was used once and discarded.

### Culture of primary sensory neurons

All mouse procedures were reviewed and approved by the University of Iowa Office of Institutional Animal Care and Use Committee. Timed pregnant mice were purchased from Charles River Laboratory. Dorsal root ganglia (DRGs) were dissected from Embryonic Day (E)13.5 mouse embryos from both male and females then seeded on plates coated with poly-d-lysine and laminin. DRG sensory neurons were incubated in Neurobasal media (Invitrogen) supplemented with B27 (Invitrogen), 50 ng/ml recombinant beta nerve growth factor (Proteintech), and 1 mM 5-fluorodeoxyuridine/1 mM uridine (Thermo Fisher Scientific) to eliminate mitotic, non-neuronal cells. Neurons were maintained at 37°C and 5% CO^2^ for the duration of each experiment.

### Cell lysis and Western immunoblotting

DRGs were lysed in RIPA buffer (50 mM Tris–HCl, 1 mM EDTA, 1% Triton X-100, 0.5% sodium deoxycholate, 0.1% sodium dodecyl sulfate, and150 mM NaCl), pH 7.4, supplemented with phosphatase inhibitors and protease inhibitors (Thermo Fisher Scientific). Cell extracts were centrifuged at 5,000 × *g* for 5 min to pellet debris and the supernatant added to Laemmli buffer with fresh β-mercaptoenthanol. Samples were separated by denaturing polyacrylamide gel electrophoresis and transferred onto nitrocellulose for Western immunoblotting with antibodies listed above. Western immunoblots were quantified in ImageJ with signals normalized internally to the Tuj1 load control. Total protein was detected with Revert prior to Western immunoblotting following manufacturer' recommendations.

### TS analysis of new protein synthesis

DRGs seeded in 96-well plates were transduced with the lentiviruses expressing the following on day in vitro (DIV) 2: TS, Bcl-xL, myristoylated mScarlet, and YARS–CMT variants. On DIV 7 cells were treated with 1 µM danoprevir to inhibit NS3 protease activity in the TS reporter and fluorescence images collected with an automated microscope (either Cytation 5 or Lionheart) every hour for 13 h. Analysis was performed with ImageJ. Briefly, time-lapse images from the same field were assembled into a stack. We performed rolling ball background subtraction then fluorescence intensity quantified from individual cells for each timepoint. TS fluorescence for individual cells was calculated at each timepoint as a ratio of fluorescence intensity measured from that cell at 1 h post-danoprevir addition because of a shift in the visual plane during image acquisition from 0 to 1 h. We quantified TS fluorescence from at least 10 cells per experimental replicate (from different wells) and at least 30 total cells from DRG preparations generated from independent mouse litters.

### Detection of activated caspase3/7

Caspase activity was visualized with CellEvent Caspase-3/7 detection reagent (Invitrogen) following manufacturer's suggestions. Briefly, DRG neurons expressing G41R-YARS or a control were incubated with the Caspase 3/7 detection reagent on DIV 4 or DIV 6, then images were collected with an automated microscope over a 12 h period. We noticed that caspase positivity would be transient and often disappear when the plasma membrane ruptured which could lead to false-negative findings. To circumvent this technical limitation, we counted the total number of positive cells observed in this 12 h period and report this as the percentage of caspase positive from an experimental condition.

### O-Propargyl-puromycin (OPP) labeling of newly synthesized protein

DRG neurons seeded in 96-well dishes were transduced with lentivirus expressing myristoylated mScarlet and Bcl-xL on DIV 2. Lentivirus expressing the CMT–YARS or an empty vector was applied on DIV 4. We labeled newly synthesized proteins using reagents and procedures from an OPP Protein Synthesis Assay kit from Vector Laboratories with the following modifications. On DIV 8, we applied OPP by performing a half-media change with fresh media containing OPP (20 µM). Some wells underwent a media change with fresh media lacking OPP to determine background. Other positive control wells were pretreated with 25 µg/ml cycloheximide. Cells were incubated with OPP-containing media for 10 min; then media were replaced once with cold PBS and again with cold PBS plus 3.7% formaldehyde. Click chemistry was performed following manufacturer's recommendations and images collected with a Lionheart FX. Fluorescence intensity was measured from at least 50 cells from three wells per experimental replicate.

### Replating DRGs for analysis of axon outgrowth and TS fluorescence

DRGs were densely seeded on 12-well plates. On DIV 2, DRGs were transduced with lentiviruses expressing myristoylated mScarlet, Bcl-xL, and conditional YARS lentivirus (empty vector, WT-YARS, G41R-YARS). For protein synthesis studies, TS lentivirus was added on DIV 5. On DIV 8, cells were dissociated from the plate with trypsin (0.05% EDTA-Invitrogen), triturated with a plastic pipette, washed in normal media, and seeded onto a new plate. For regrowth and TS studies, cells were seeded in 96-well plates. For protein biochemistry, cells were immediately lysed or seeded in 12-well plates and lysed 24 h later. For regrowth studies, myristoylated mScarlet images were collected every hour in an automated microscope. Images were analyzed with a custom ImageJ macro that auto-contrasts and thresholds each image then measures axon particles as total axon area (TAA) from the binarized image. This procedure eliminates most signal from the cell body due to high focal fluorescence. Axon regrowth was visualized over 11 h with automated microscopy and quantified by measuring TAA at a given timepoint normalized to TAA at time 1 h. At least 10 image fields were measured over time per condition per replicate. For each timepoint, the TAA in a given image field was calculated as a ratio of time 1 h TAA from that field. Axonal regrowth measurements for at least 30 fields per condition were calculated this way and averaged respective to experimental replicate. In pharmacology studies, drugs were applied immediately after replating and left in the media during the duration of the experiment. Protein synthesis experiments were conducted with the TS reporter following procedures described above.

### Experimental design and statistical analysis

Experimental replicates represent sensory neuron cultures derived from independent mouse litters and at least two independent lentiviral preparations. Sex is not evaluated as a biological variable as we combine DRGs isolated from both male and female embryos. For 96-well studies, we include intraexperimental replicates and use the average of these internal replicates to generate one experimental replicate. Sample sizes have enough power to identify statistically significant differences without overpowering. Statistical analyses were performed in GraphPad Prism. We assess normality in datasets and use a one-way ANOVA with post hoc tests with Tukey's correction for multiple comparisons when data meet criteria of a normal distribution. Otherwise, we employ nonparametric tests such as the one-sample Wilcoxon test or Kruskal–Wallis analysis with Dunn's post hoc test when multiple conditions are assessed. Specific statistical tests employed for each experiment are described with the accompanying figure legend.

## Results

### CMT mutations in tyrosyl-tRNA synthetase (YARS) provoke axon degeneration and sensory neuron death

Autosomal dominant mutations in human YARS cause DI-CMTC, and introducing YARS–CMT variants into a wild-type background is sufficient to induce peripheral neuropathy in most model systems (Niehues et al., 2015; Bervoets et al., 2019; Zuko et al., 2021). For this study we used embryonic-derived mouse sensory neurons isolated from DRGs. These neurons are readily manipulated with lentiviruses and regrow severed axons which enabled us to assess how YARS–CMT variants impact axon regrowth. We prepared lentivirus expressing human YARS constructs downstream of the ubiquitin promoter. An empty vector and wild-type YARS were used as controls. We generated lentiviruses expressing three different variants identified in DI-CMTC populations, G41R, D81I, and 153–156delVKQV(Δ153–156; Jordanova et al., 2003, 2006; Hyun et al., 2014). We intended to include another well-studied variant, E196K; however, we consistently observed poor protein expression and did not pursue this variant further.

**Figure 1.**
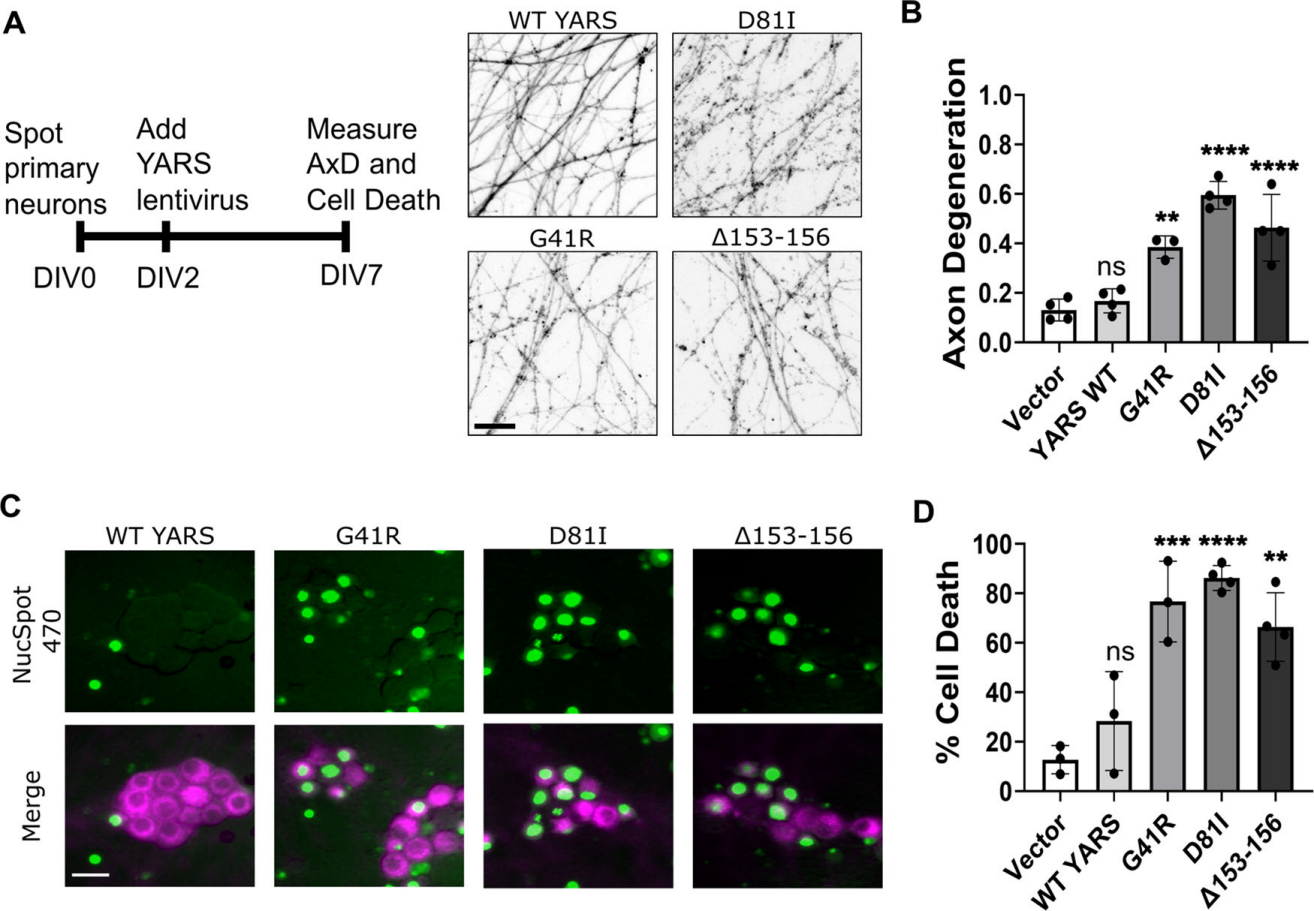
CMT-linked variants of tyrosyl-tRNA synthetase (YARS) induce caspase-dependent cell death and axon degeneration. ***A***, Primary sensory neurons from embryonic DRG were transduced with lentivirus expressing the indicated YARS variant on DIV 2. Axon fragmentation was apparent by DIV 7 from neurons expressing YARS variants with CMT-linked mutations with quantification shown in ***B***. ***C***, YARS–CMT expressing neurons also stain positive for the cell-impermeable dye NucSpot which enters cells with compromised membrane permeability and fluoresces in complex with DNA in the nucleus. Cell membranes are labeled with exogenously expressed myristoylated-mScarlet (magenta). Quantification is shown in ***D*** (*N* = 3–4). Scale bar, 20 µm. Error bars indicate ±1 SD. For statistical tests, one-way ANOVA was performed with post hoc *t* tests where *p < 0.05, **p < 0.01, ***p < 0.005, and ****p < 0.001. ns, not significant.

We first evaluated whether prolonged expression of CMT–YARS variants affected axon integrity. Lentiviral preparations were applied to cultured DRGs at DIV 2. On DIV 7, the degree of axon degeneration was quantified using an ImageJ macro that measures particle circularity to delineate fragmented versus intact axon area to calculate a “degeneration index” from 0 to 1 (least to most degeneration, respectively; Gerdts et al., 2011). DRG neurons expressing any YARS–CMT variants displayed significant axon degeneration compared with empty vector control (Fig. 1*A*,*B*). Importantly, WT-YARS expression did not provoke axon degeneration, indicating that elevated levels of tyrosyl-tRNA synthetase did not underlie the observed axon degeneration (Fig. 1*A*,*B*). At the same timepoint, we also observed widespread cell death (>60%) in all three mutant conditions as measured by incorporation of the membrane impermeable dye, NucSpot 470 (Fig. 1*C*,*D*).

**Figure 2.**
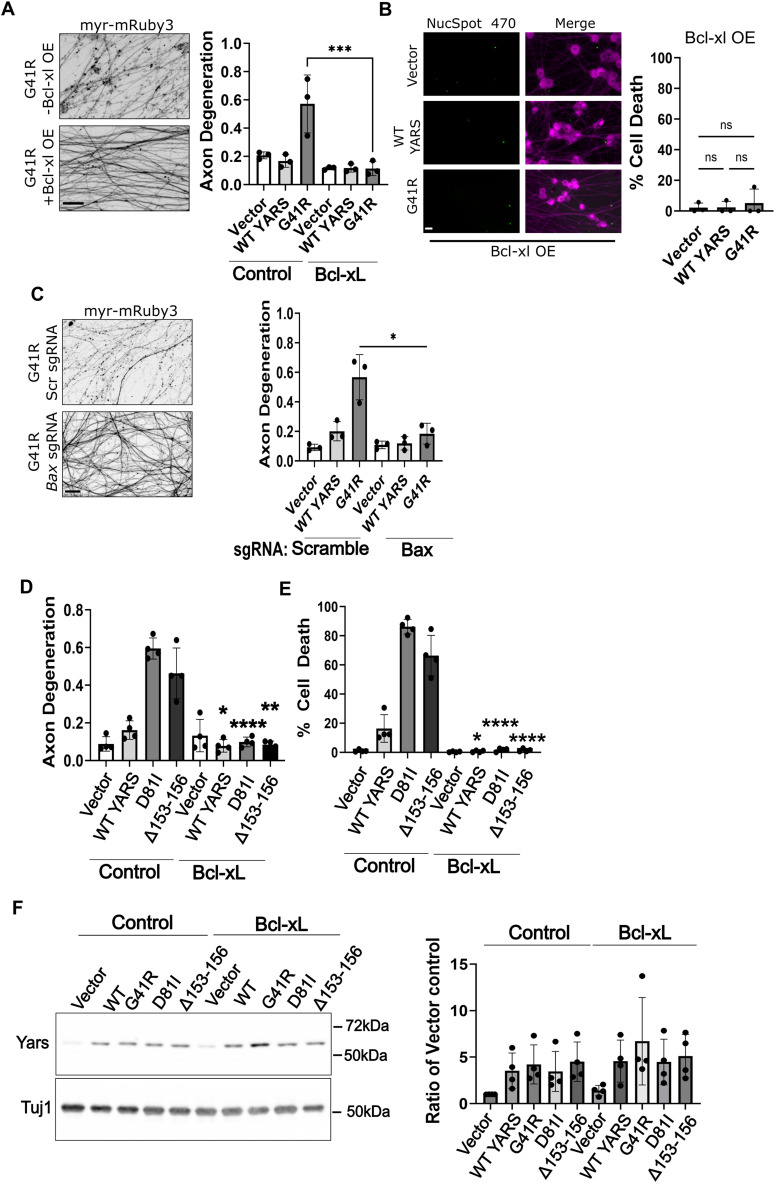
CMT–YARS induces axon degeneration and cell death apoptosis. Cells were transduced with YARS-expressing lentivirus and a lentivirus expressing the antiapoptotic protein Bcl-xL. ***A***, ***B***, Expressing Bcl-xL suppressed axon degeneration (***C***) and cell death induced by YARS-G41R (*N* = 3). ***C***, Crispr inactivating the proapoptotic protein BAX also suppressed axon degeneration in G41R-expressing neurons compared with scrambled (scr) sgRNAs (*N* = 3). ***D***, ***E***, Bcl-xL expression suppressed cell death induced by YARS–CMT variants D81I and Δ153–156 (*N* = 4). ***F***, Western blot analysis of endogenous YARS protein levels from DRG neurons expressing the indicated YARS–CMT variant with quantification (*N* = 4). Scale bar, 20 µm. Error bars indicate ±1 SD. For statistical tests, one-way ANOVA was performed with post hoc *t* tests where *p < 0.05, **p < 0.01, and ****p < 0.001. In ***C*** and ***D***, asterisks identify significant differences after a post hoc *t* test comparing cell death for an individual YARS variant with or without Bcl-xL.

Caspase-dependent apoptosis promotes neuronal loss during development and in the context of neurodegeneration. Inhibiting caspase activation by Bcl-xL overexpression or CRISPR inactivating BAX suppressed cell death and axon degeneration in neurons expressing YARS-G41R (Fig. 2*A*–*C*) as well as D81I-YARS and Δ153–156-YARS (Fig. 2*D*,*E*). Bcl-xL expression did not affect CMT–YARS protein levels (Fig. 2*F*) indicating neuroprotective effects were due to this protein's antiapoptotic function rather than downregulation of mutant CMT–YARS expression.

### CMT–YARS reduces protein synthesis in DRG sensory neurons

**Figure 3.**
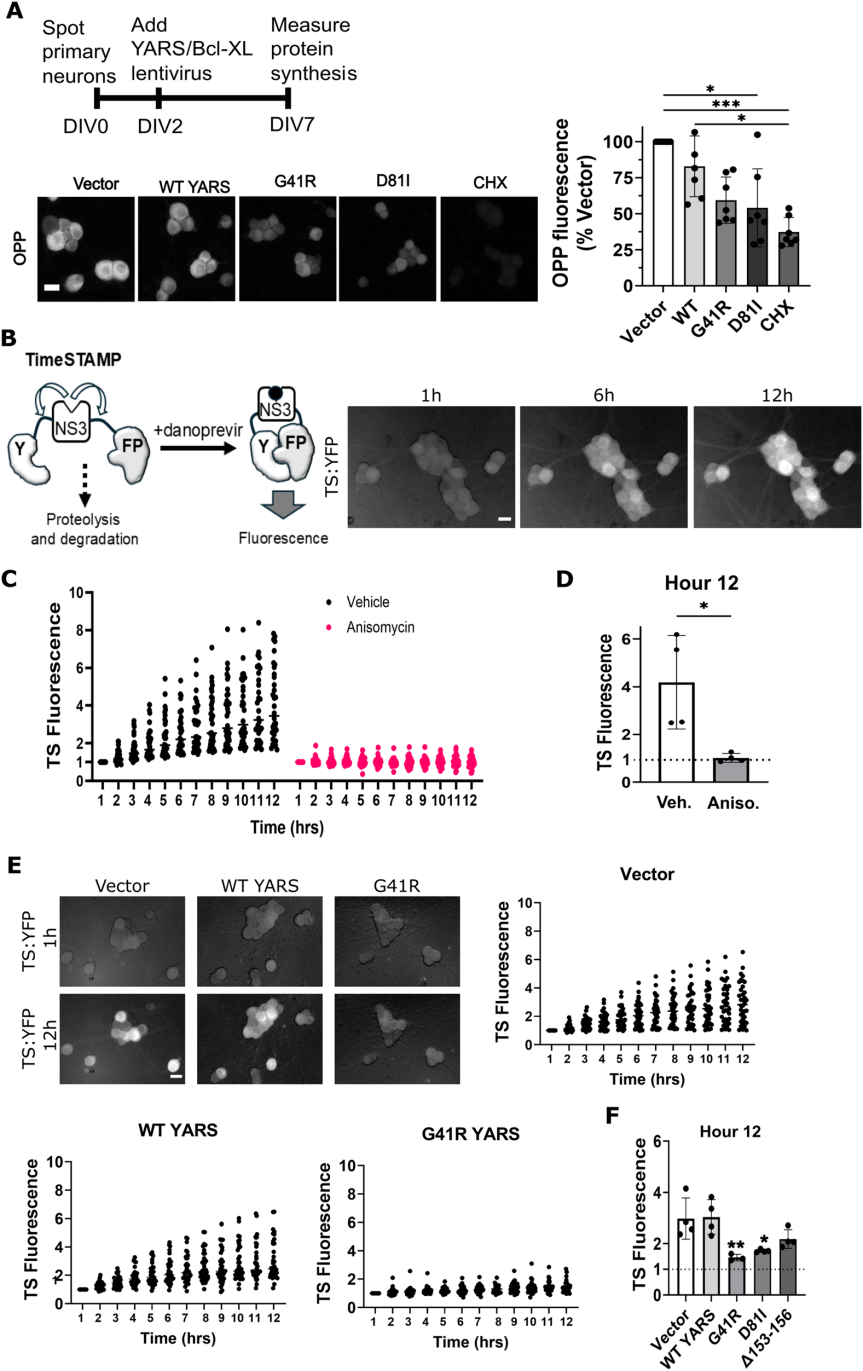
CMT–YARS expression reduces protein synthesis. DRG sensory neurons were transduced on DIV2 with the indicated YARS lentivirus and Bcl-xL lentivirus to suppress caspase activation. ***A***, Newly synthesized proteins were labeled with OP for 10 min and visualized after click chemistry with AZdye488. OPP fluorescence was reduced in the presence of G41R- or D81I-YARS compared with empty vector or wild-type YARS. Applying the protein synthesis inhibitor cycloheximide (CHX) prior to labeling further reduced protein synthesis. Representative images and quantification (*N* = 6) are shown. ***B***, Diagram of TS reporter for newly synthesized proteins. Steady-state TS fluorescence is low as newly synthesized reporter undergoes proteolysis and protein degradation. Applying the NS3 inhibitor danoprevir stabilizes newly synthesized TS protein, split YFP fragments assemble, and fluorescence increases over time. On the right, representative images show TS fluorescence after danoprevir application. ***C***, TS fluorescence was measured once per hour during a 12 h time frame from individual cells across four experimental replicates after danoprevir addition and represented as a ratio of the 1 h timepoint. In ***D***, the change in fluorescence at 12 h was averaged in four independent experiments (from at least 40 cells). Applying the translation inhibitor anisomycin (10 µM) suppresses TS fluorescence after danoprevir treatment (***C*** and ***D***). ***E***, G41R-YARS reduces TS fluorescence after danoprevir treatment compared with an empty vector or WT-YARS (*N* = 4) with 12 h time points shown in ***F*** from experiments performed with all three CMT–YARS variants. Extended Data Figure 3-1 display additional TS fluorescence data with D81I and Δ153–156 and Western blot analysis. Scale bar, 20 µm. Error bars indicate ±1 SD. A Kruskal–Wallis analysis was performed for data shown in ***A*** with Dunn's post hoc test for multiple comparisons where *p < 0.05 and ***p < 0.005. For experiments in ***D*** and ***F***, a one-way ANOVA was performed with post hoc *t* tests where *p < 0.05, **p < 0.01, and ***p < 0.005.

We next evaluated whether CMT–YARS variants affect protein synthesis in sensory neurons. We overexpressed Bcl-xL via lentiviral transduction to prevent caspase activation and circumvent changes in protein synthesis expected downstream of apoptosis. YARS variants G41R and del153 reduce aminoacylation activity in vitro and in vivo (Niehues et al., 2015) while activity of the D81I variant has not been determined. We used two strategies to evaluate protein synthesis in DRG neurons expressing these variants. We first labeled newly synthesized proteins with the amino acid analog OPP and then used click chemistry to visualize this analog with the fluorescent probe AZdye 488. DRGs transduced with G41R- or D81I-YARS showed a modest decrease in OPP fluorescence compared with empty vector controls, with statistically significant differences only observed between YARS D81I and the empty vector condition (Fig. 3*A*). The translation inhibitor cycloheximide did not completely suppress OPP fluorescence, indicating off-target fluorophore labeling during the click chemistry reaction which would decrease sensitivity of the assay. Additionally, OPP labeling requires cell fixation, limiting our investigation to a single timepoint and preventing analysis of the temporal dynamics of protein synthesis.

10.1523/ENEURO.0337-25.2026.f3-1 Figure 3-1 **Reduced protein synthesis in sensory neurons expressing CMT-YARS. (A) & (B)** TS fluorescence from individual cells after danoprevir addition expressing D81I-YARS (28 cells from three independent experiments) or Δ153-156YARS (36 cells from 3 independent experiments). **(C)** Western blot of DRG extracts four hours after danoprevir addition. TS was detected with an antibody to GFP = with quantification on the right (N = 4). Error bars represent +/-1 SD. We performed a Kruskal-Wallis analysis with Dunn’s post-hoc test to assess significance where ** p˂0.01, and ***p˂0.005. Download Figure 3-1, TIF file.

As an alternative approach to measure protein synthesis in live cell cultures, we leveraged a genetically encoded fluorescent reporter for protein synthesis called TimeSTAMP, herein named TS (Lin et al., 2008; Lin and Tsien, 2010). The TS construct consists of the nonstructural protein 3 (NS3) protease flanked by two linker regions and split yellow fluorescent protein (YFP) fragments. The protein reporter is continuously synthesized and degraded, as the NS3 protease targets cleavage sites in the adjacent linker regions. Applying an NS3 protease inhibitor (danoprevir) prevents degradation of newly synthesized TS protein, allows assembly of the split YFP fragments, and the subsequent change in fluorescence serves as a readout for new protein synthesis (Fig. 3*B*). DRGs were transduced with lentivirus expressing TS on DIV 5 and danoprevir added on DIV 8. Over the next 12 h, fluorescence in individual neurons was tracked once an hour with an automated microscope; then each neuron's fluorescence signal was normalized to its value at 1 h post-danoprevir treatment (Fig. 3*C*). Fluorescence intensity increased over time however the rate varied from neuron-to-neuron (Fig. 3*C*), consistent with other single-cell studies of protein synthesis (Han et al., 2014). We limited our statistical analysis to the relationship between conditions at the final timepoint with relative fluorescence intensities averaged from at least 10 cells per experimental replicate (evaluating at least 30 total cells per condition). Importantly, treatment with the translation inhibitor anisomycin suppressed the increase in fluorescence, confirming this signal represents newly synthesized TS (Fig. 3*C*,*D*).

DRGs expressing any CMT–YARS variant displayed substantial defects in protein synthesis at DIV 8 compared with Vector and WT-YARS conditions (Fig. 3*E*,*F*; Extended Data Fig. 3-1*A*,*B*). Δ153–156-YARS expression caused a milder synthesis defect, with severity falling between negative controls and G41R-/D81I-YARS (Extended Data Fig. 3-1). The decrease in TS fluorescence was confirmed by Western blot from whole cell extracts for the TS construct (Extended Data Fig. 3-1).

**Figure 4.**
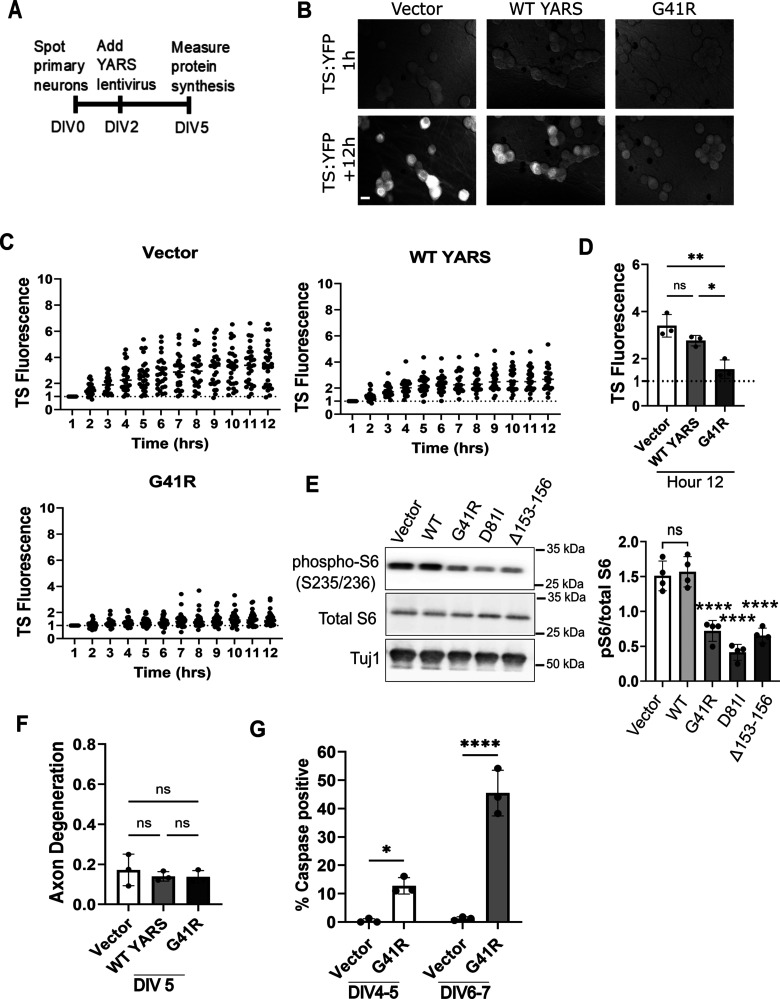
Reduced protein synthesis precedes axon degeneration in neurons expressing G41R-YARS. ***A***, We measured protein synthesis in neurons expressing G41R-YARS on DIV5. ***B***, ***C***, TS fluorescence after danoprevir addition in neurons transduced with lentivirus expressing G41R-YARS, WT-YARS, or an empty vector over a 12 h time period with TS fluorescence at 12 h in ***D*** (*N* = 3). ***E***, Representative Western blot of phosphorylated S6 ribosomal protein from CMT–YARS-expressing neurons with quantification of phospho-S6 as a ratio of total S6 protein (*N* = 4). ***F***, Axon degeneration on DIV 5. ***G***, We visualized caspase3/7 activation over a 12 h time period with a cell-permeable fluorescent dye that translocates to the nucleus and fluorescence in the presence of activated caspases. We noted positive caspase labeling was transient in a dying population, so we tracked the total number of caspase-positive cells over a 12 h interval. A small, though significant population of caspase-positive cells were detected in the presence of G41R-YARS between DIV 4 and DIV 5. This percentage increased between DIV 6 and DIV7. Scale bar, 20 µm. Error bars indicate ±1 SD. For statistical tests, one-way ANOVA was performed with post hoc *t* tests where *p < 0.05, **p < 0.01, and ***p < 0.005.

We hypothesized the protein synthesis defect on DIV 8 is an early outcome of CMT–YARS expression and precedes onset of apoptosis. To evaluate this further, we measured protein synthesis on DIV 5, 3 d after viral transduction (Fig. 4*A*). Even at this early timepoint, G41R-YARS expression caused a protein synthesis defect compared with empty vector and WT-YARS controls (Fig. 4*B*–*D*). We also detect a significant decrease in levels of phosphorylated S6 ribosomal protein when YARS–CMT variants are expressed (Fig. 4*E*), indicative of reduced protein synthesis. In the absence of Bcl-xL, we observe no axon degeneration at this timepoint (Fig. 4*F*), and caspase 3/7 activity was detected in 12% of G41R-expressing neurons, lower than the 40% caspase3/7 positive neurons observed 2 d later (Fig. 4*G*). Therefore, G41R-induced defects in protein synthesis occur days before axon fragmentation and widespread induction of caspase activation.

### Investigating effects of CMT–YARS on axon regeneration

**Figure 5.**
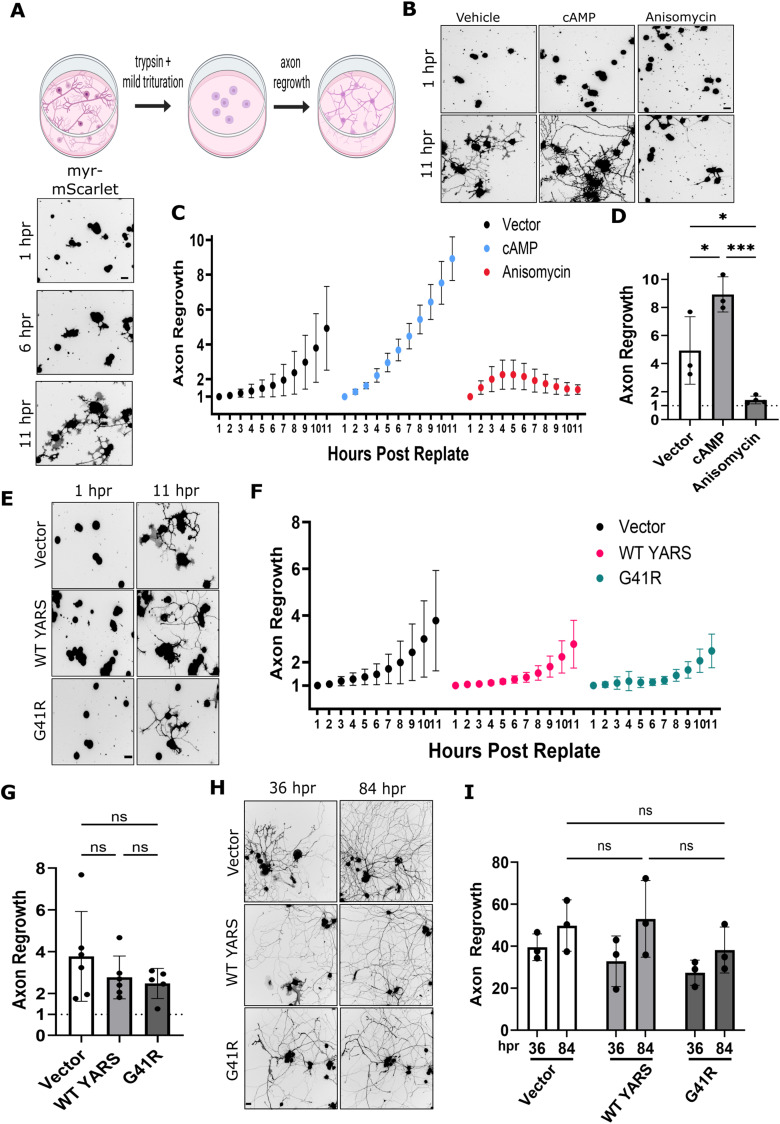
G41R-YARS does not impair axon regeneration after replating. ***A***, Sensory neurons were transduced with YARS lentiviruses as described in prior experiments as well as Bcl-xl expressing lentiviruses to suppress caspase-dependent cell death. On DIV 8, neurons were lifted from the plate with trypsin digestion, axons sheared off by mild trituration, and neurons seeded onto new plates. Axon regrowth was visualized over 11 h with automated microscopy. We quantified “axon regrowth” as the axon area at a given timepoint normalized to axon area at time 1 h. Example images are shown below. ***B***, ***C***, Applying a cell-permeable cAMP analog (50 µM) accelerated axon regrowth, while the protein synthesis inhibitor anisomycin (10 µM) suppressed axon regrowth. ***D***, Axon regrowth in neurons treated with cAMP or anisomycin at 11 hpr (*N* = 3). ***E***–***G***, Axon regrowth in G41R-YARS neurons was unchanged compared with WT-YARS or an empty vector with 11 hpr (*N* = 5–6). ***H***, ***I***, Axon regrowth in G41R-YARS neurons was similar to control conditions at 36 hpr. A slight reduction in axon area was noted at 84 hpr in G41R-YARS neurons; however, this difference did not reach statistical significance (*N* = 3). Scale bar, 40 µm. Error bars indicate ±1 SD. For statistical tests, one-way ANOVA was performed with post hoc *t* tests where *p < 0.05, **p < 0.01, and ***p < 0.005. Replating diagram was generated with BioRender.

Axon outgrowth during development and regeneration depends on new protein synthesis. Introducing CMT–YARS by lentivirus on DIV 2 precluded us from assessing its effects on early axon outgrowth during DIV 0–1. To circumvent this limitation, we utilized a replating protocol to remove axons and stimulate axon regeneration (Frey et al., 2015; Fig. 5*A*). CMT–YARS and Bcl-XL were transduced into DRG sensory neurons as described above. On DIV 8, DRG cultures were briefly trypsinized to detach cells from the plate. The collected DRG suspensions were triturated and resuspended to shear off the axons and then reseeded onto a new plate. Replated DRGs sprout new axons with growth cones within the first few hours after replating (Fig. 5*A*). Consistent with prior studies activating adenylate cyclase with forskolin, applying a cell-permeable cyclic-AMP analog accelerated axon regeneration (Kilmer and Carlsen, 1984; Cai et al., 1999; Neumann et al., 2002; Qiu et al., 2002; Ghosh-Roy et al., 2010; Frey et al., 2015; Hao et al., 2016; Fig. 5*B–D*). Conversely, anisomycin treatment inhibited regeneration, confirming axon regrowth following replating is dependent on new protein synthesis (Twiss and Shooter, 1995). Similar to TS studies in Figures 3 and 4, we limited our statistical analysis to the relationship between conditions at the final timepoint of 11 h postreplating (hpr; Fig. 5*D*). Unexpectedly, the rate of axon regrowth from replated DRGs expressing G41R was similar to regrowth from empty vector and WT controls (Fig. 5*E*–*G*). We tracked axonal growth up to 36 and 84 hpr and observed a modest, nonsignificant decrease in G41R-expressing cells at 84 hpr (Fig. 5*H,*I).

**Figure 6.**
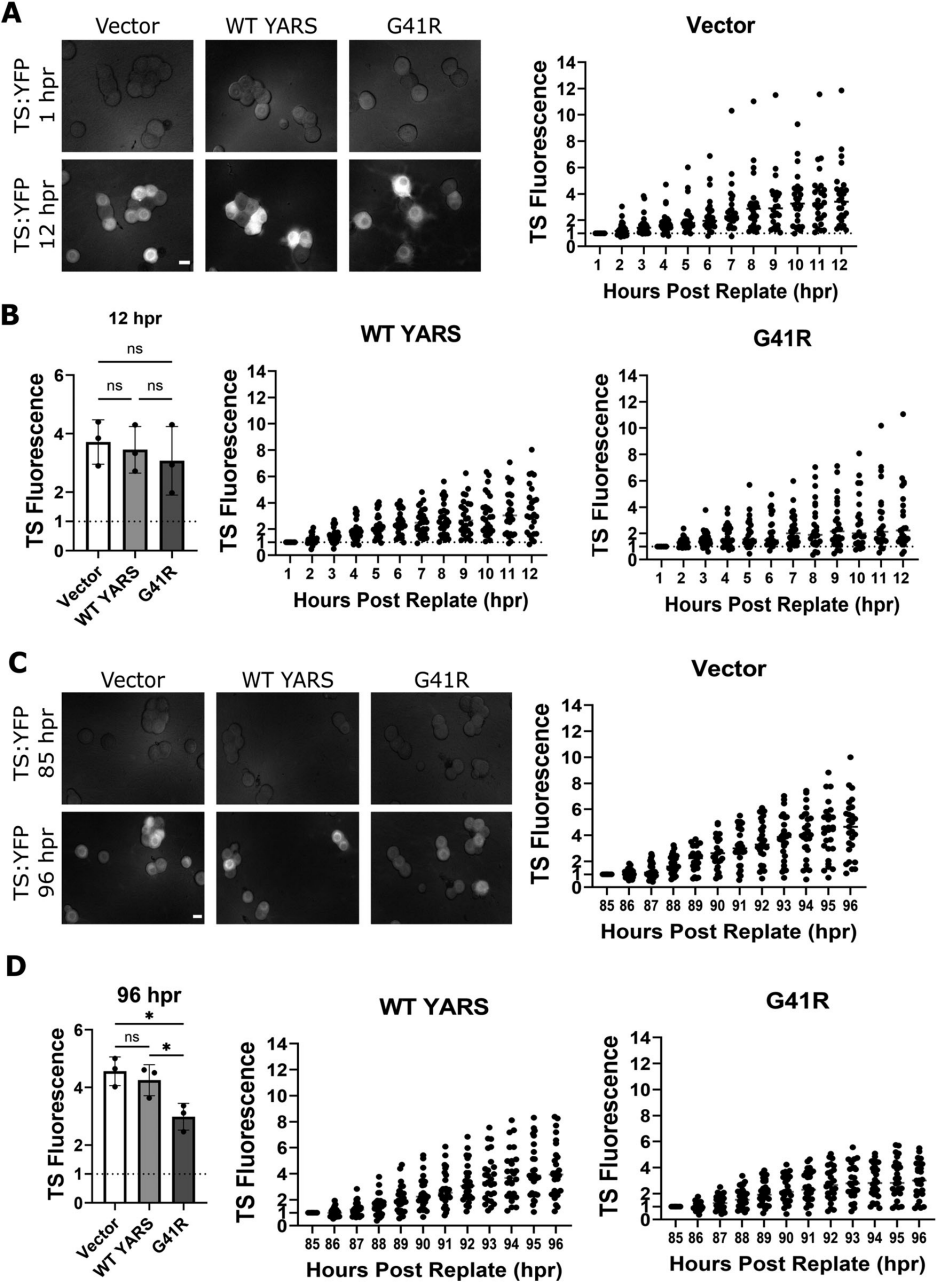
Replating rescues protein synthesis in G41R-YARS neurons. ***A***, TS fluorescence was measured in replated neurons transduced with lentivirus expressing G41R-YARS, WT-YARS, or an empty vector. ***B***, TS fluorescence at 12 h post-danoprevir application in replated neurons (*N* = 3). ***C***, Neurons 84 h after replating were treated with danoprevir and TS fluorescence tracked over 12 h with terminal fluorescence at 96 hpr shown in ***D*** (*N* = 3). Extended Data Figure 6-1 demonstrates that axotomy is not sufficient to rescue protein synthesis in YARS-G41R neurons. Scale bar, 20 µm. Error bars indicate ±1 SD. For statistical tests, one-way ANOVA was performed with post hoc *t* tests where *p < 0.05, **p < 0.01, and ***p < 0.005.

Normal axon regeneration in G41R-expressing neurons prompted us to investigate new protein synthesis. We measured TS fluorescence immediately after replating over a 12 h period. To our surprise, protein synthesis in replated, G41R-expressing neurons was unchanged at 12 h compared with empty vector and WT-YARS (Fig. 6*A*,*B*). We suspected rescue of protein synthesis might subside over time as replated neurons mature. We tested this prediction by incubating replated neurons for 84 h before adding danoprevir and measuring TS fluorescence over the next 12 h (96 hpr). While TS synthesis in the G41R condition initially appeared normal compared with vector and WT-YARS, synthesis tapered off and by 96 hpr was significantly reduced compared with empty vector and WT-YARS (Fig. 6*C,*D). Therefore, replating temporarily alleviates protein synthesis defects present in G41R-YARS neurons. Axotomy stimulates regeneration in PNS neurons, and we predicted that severing distal axons would also rescue protein synthesis. We performed axotomy in nonreplated DRG neurons expressing G41R-YARS at DIV 8; however, severing axons was not sufficient to rescue protein synthesis (Extended Data Fig. 6-1).

10.1523/ENEURO.0337-25.2026.f6-1 Figure 6-1 **Axotomy is not sufficient to restore protein synthesis in YARS-G41R sensory neurons. (A)** DRG sensory neurons seeded in a spot culture were transduced on DIV2 with lentivirus expressing an empty vector or YARS-G41R, Bcl-xL to prevent caspase activation, and TimeSTAMP. On DIV8 a razor was used to sever axons around the spot culture. One hour later, danoprevir was added and TimeSTAMP visualized over the next twelve hours. Data points from individual cells are shown for each condition (from at least 40 cells per condition in four independent replicates) with the twelve-hour time point **(B)**. Error bars represent +/-1 SD. For statistical tests, one-way ANOVA was performed with post-hoc t-tests where *p˂0.05. Download Figure 6-1, TIF file.

**Figure 7.**
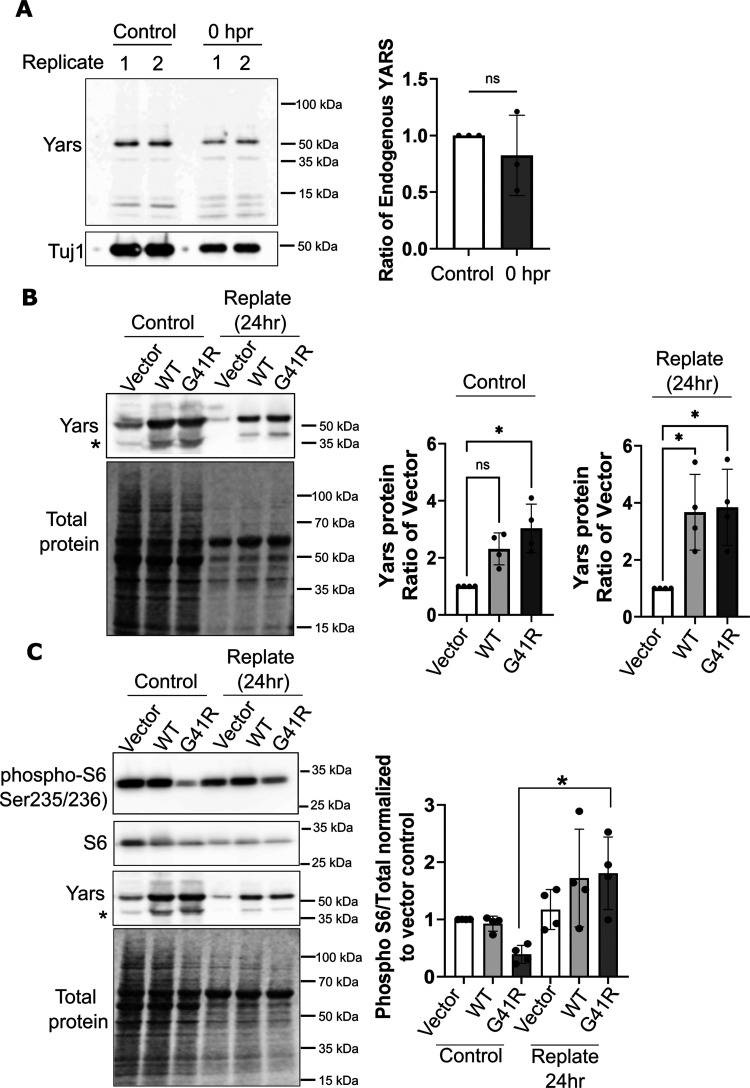
Replating does not alter expression of endogenous or exogenously expressed YARS proteins. ***A***, Whole cell extracts were collected from neurons on DIV 8 as naive controls or immediately after replating (0 h). Endogenous YARS protein levels were detected by Western immunoblotting and normalized to Tuj1 as a load control. Two replicates are shown in the representative Western blot from both conditions. Extended Data Figure 7-1 shows unchanged levels in endogenous YARS as well as YARS-mScarlet by Western blot and fluorescence. ***B***, YARS Western blot from DRGs expressing an empty vector, WT-YARS, or G41R-YARS in nonreplated controls or 24 hpr. Revert stain was used to detect total protein. For quantification, we compared the ratio of overexpressed YARS to endogenous YARS within controls or replated samples. ***C***, Western blots for phosphorylated S6 ribosomal protein in samples from nonreplated controls or 24 hpr with quantification of phospho-S6 normalized to total S6 protein and displayed as a ratio to levels in vector control. Since total protein is consistently lower in replated neurons, phospho-S6 levels were increased though bands appear similar when compared side-by-side with nonreplated controls. Asterisk identifies a YARS-positive band that represents a proteolysis event or posttranslational modification. Error bars indicate ±1 SD. Statistical comparisons were assessed with Kruskal–Wallis test with Dunn's post hoc test for multiple comparisons where *p < 0.05. ns, not significant.

We examined protein levels of endogenous YARS and lentiviral-expressed G41R-YARS after replating to ascertain whether changes in TS synthesis were due to alterations in protein levels. For example, replating might upregulate endogenous YARS and suppress deleterious effects of G41R-YARS. We first compared endogenous YARS which was unchanged in prereplated samples compared with those collected immediately after replating (Fig. 7*A*) or 24 hpr (Extended Data Fig. 7-1). We noted a steep decrease in Tuj1 protein from replated samples, possibly due to cell death, and relied on a total protein stain for samples collected 24 hpr.

10.1523/ENEURO.0337-25.2026.f7-1 Figure 7-1 **YARS-mScarlet expression in DRGs. (A)** We compared the ratio of endogenous YARS protein in DRGs pre-replating (pre-rep) to twenty-four hours post replating (hpr). Two replicates are shown in the representative western blot with quantification. **(B)** We compared endogenous YARS protein to overexpressed, mScarlet-tagged YARS protein after replating by western immunoblotting. The ratio of YARS (G41R)-mScarlet to endogenous YARS was still elevated after replating at a time point when protein synthesis defects were reversed. Asterisk identifies a YARS-positive band below mScarlet-tagged proteins that are likely proteolysis events. **(C)** Example images of high lentiviral transduction rate in DRG sensory neurons expressing wildtype YARS-mScarlet or YARS (G41R)-mScarlet. Images were collected with an automated microscope as a montage then stitched together to provide a population-wide view of mScarlet expression with Hoechst 33342 labeling nuclei. **(D)** Stitched images of replated neurons expressing wildtype YARS-mScarlet or YARS (G41R)-mScarlet to demonstrate expression in a majority of cells after replating when protein synthesis defects are reversed. Error bars represent +/-1 SD. Scale bar = 200 µm. Download Figure 7-1, TIF file.

We next evaluated protein levels of exogenously expressed YARS 24 hpr to determine if this procedure reduced G41R-YARS expression. When compared with endogenous YARS, replating did not reduce protein levels of exogenously expressed WT or G41R-YARS (Fig. 7B). DRGs were also transduced with a version of WT-YARS and G41R-YARS tagged with mScarlet to distinguish overexpressed YARS levels with endogenous YARS on a Western blot. WT-YARS-mScarlet and G41R-mScarlet expression levels were nearly eightfold higher than endogenous YARS in the same lysate collected 24 hpr after replating when protein synthesis defects are substantially diminished (Extended Data Fig. 7-1), similar to expression levels observed in nonreplated DRGs. Therefore, replating does not rescue protein synthesis through increasing endogenous YARS or reducing exogenously expressed YARS.

Since a reduction in phosphorylated S6 ribosomal protein was detected from YARS–CMT neurons, we next determined if DRG replating rescues phosphorylation of this protein and would point to modulation of mTOR signaling. Indeed, S6 ribosomal protein phosphorylation was increased from DRG neurons collected 24 hpr (Fig. 7*C*). Phospho-S6 protein levels were normalized to total protein from each sample, which was lower in replated neurons as described above, revealing a significant increase when comparing control YARS-G41R samples to postreplated YARS-G41R samples. These data are consistent mTOR activation and protein synthesis recovery.

### Replating-induced rescue of protein synthesis requires mTOR

**Figure 8.**
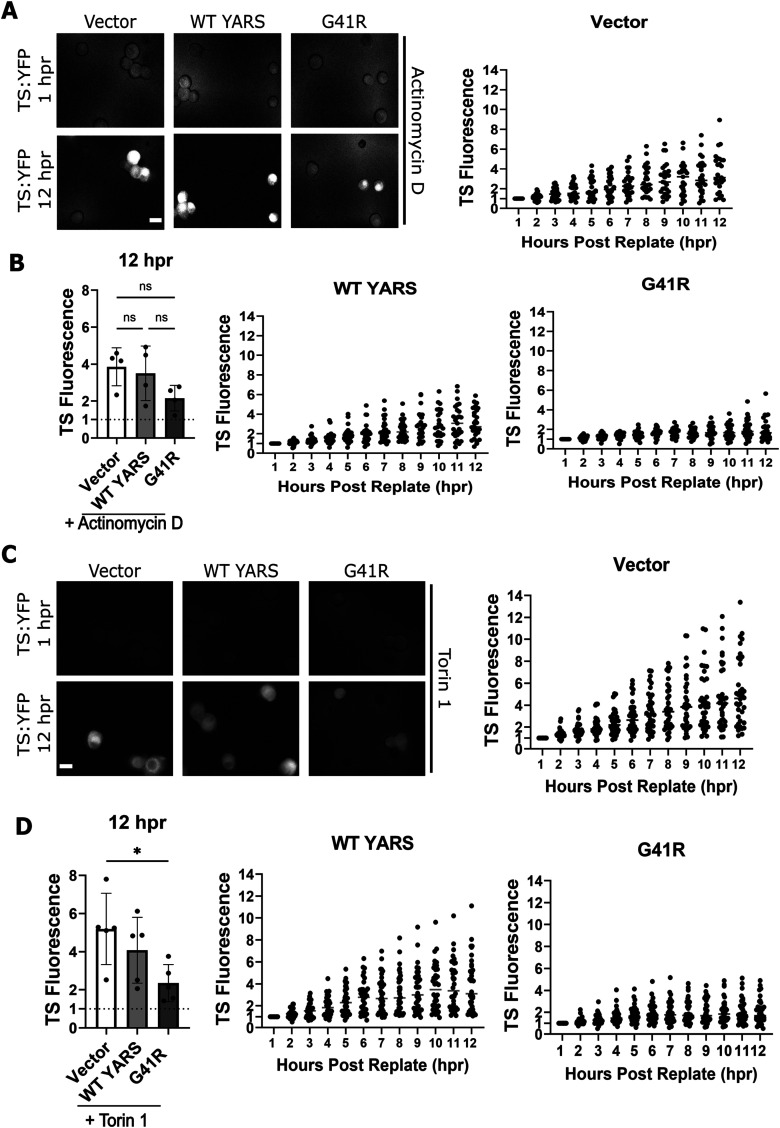
New transcription and mTOR participate in rescue of protein synthesis in replated neurons. ***A***, Replated neurons were treated with the transcription inhibitor actinomycin D (1 µg/ml), and then TS fluorescence is measured after danoprevir application. ***B***, Change in TS fluorescence in individual cells through 12 h postapplication (*N* = 4). ***C***, Replated neurons were pretreated with Torin 1 (150 nM), and then TS fluorescence is measured after danoprevir application. ***D***, Change in TS fluorescence in individual cells through 12 h postapplication (*N* = 5). Error bars indicate ±1 SD. For statistical tests, one-way ANOVA was performed with post hoc *t* tests where *p < 0.05. ns, not significant.

Replating stimulates transcriptional upregulation of regeneration-associated genes (RAGs; Mahar and Cavalli, 2018; Tome and Almeida, 2024). To test whether new transcription is required for rescuing protein synthesis in G41R neurons, we applied the transcription inhibitor actinomycin D to replated DRGs and measured TS fluorescence over the next 12 h. In the presence of actinomycin D, TS synthesis was delayed in G41R-expressing neurons at early timepoints after danoprevir addition (Fig. 8*A*); however, no statistically significant differences were noted at the 12 h time point (Fig. 8*B*). Actinomycin D treatment did not reduce TS fluorescence in empty vector or WT-YARS conditions suggesting reduced synthesis in G41R-YARS was not due to a global impairment in TS synthesis triggered by inhibiting transcription. These results indicate new transcription contributes to the restoration of protein synthesis in G41R-YARS neurons after replating, though other factors likely participate in this effect.

Axon injury stimulates mTOR, a well-established regulator of protein synthesis that promotes axon regeneration (Park et al., 2008; Abe et al., 2010; Terenzio et al., 2018; Koley et al., 2019). We treated neurons with Torin 1, an inhibitor of both mTORC1 and mTORC2 after replating. Torin1 treatment caused a reduction in TS synthesis in replated G41R-expressing neurons compared with WT or empty vector conditions (Fig. 8*C*,*D*). Therefore, the mTOR pathway is necessary for rescuing protein synthesis in YARS-G41R neurons after replating.

## Discussion

AaRS supply amino acid-charged tRNAs for new protein synthesis and perturbing any one of these enzymes can have devastating consequences. For example, homozygous recessive mutations in YARS cause multisystem failure (Nowaczyk et al., 2017), while autosomal dominant mutations manifest in DI-CMTC, a peripheral neuropathy restricted to sensory and motor neurons (Jordanova et al., 2003, 2006; Hyun et al., 2014; Gonzaga-Jauregui et al., 2015). The mechanistic basis for such specificity has been a recurring question. Moreover, since CMT symptoms often emerge in early adolescence or later, how do PNS neurons successfully innervate their targets during development when the need for new protein synthesis would be especially high? We propose that upregulation of axon outgrowth pathways during PNS development insulates these neurons against CMT–YARS and the switch from development to maintenance diminishes these safeguards.

While our sensory neuron model recapitulates several key observations reported in other CMT models, caspase-dependent cell death is not noted in mouse or drosophila models of YARS–CMT. Prolonged repression of protein synthesis might be more severe in our model to the extent of triggering apoptotic cell death. We bypassed this limitation through Bcl-xL overexpression which prevents cytochrome c release and caspase activation. Under these conditions, sensory neurons tolerate prolonged CMT–YARS expression over 8 d in culture enabling us to evaluate long-term impacts on protein synthesis and conduct replating studies for axon regrowth.

Neurons expressing three different YARS alleles identified in patients with DI-CMTC reduced protein synthesis, confirming prior studies with two alleles (G41R and Δ153–156) and testing D81I for the first time. Poor expression precluded us from evaluating E196K which shows minimal loss of enzymatic activity in vitro yet does elicit neuropathy in vivo (Storkebaum et al., 2009; Froelich and First, 2011; Niehues et al., 2015). Protein synthesis defects coincided with caspase activation and were not suppressed by Bcl-xL overexpression, suggesting CMT–YARS expression triggers a decline in protein synthesis upstream of caspase activation. Our findings do not rule out gain-of-function interactions as the mechanism underlying CMT–YARS neuropathy (Blocquel et al., 2017; Bervoets et al., 2019; Ermanoska et al., 2023) which might reduce protein synthesis independent of aminoacylation activity. The integrated stress response (ISR) likely plays an important role as this pathway represses protein synthesis and genetic or pharmacological inhibition of the ISR rescues motor neuropathy in CMT mouse models (Spaulding et al., 2021). Investigating whether replating pathways intersect with the ISR would be an exciting future direction. Altogether, diminished protein synthesis would hinder neuronal function and compromise health due to depletion of short-lived proteins.

PNS cells maintain the capacity for axon regeneration into adulthood. Nerve transection activates local mTOR signaling which boosts synthesis of proregenerative factors (Tome and Almeida, 2024). Retrograde signals from the lesion site stimulate transcription of RAGs which collectively drive new axon outgrowth. mTOR activation is the most direct route toward stimulating protein synthesis; however, axotomy was not sufficient to rescue protein synthesis in YARS-G41R neurons. Replating rescues phosphorylation of S6 ribosomal protein in YARS-G41R neurons indicating additional signaling events are activated during this procedure that are likely required for synthesis rescue. Trypsin facilitates substrate detachment and might stimulate intracellular signaling through cleavage of transmembrane receptors. Axon regrowth after replating is highly branched compared with regrowth after axotomy which indicates loss of polarity or activation of a distinct gene repertoire. Though TS fluorescence in replated, YARS-G41R neurons treated with the transcription inhibitor Actinomycin D was diminished, global inhibition did not elicit a significant reversal in protein synthesis. This compound might have decayed during the timecourse as there was a notable lag in TS fluorescence upon Actinomycin D treatment. New transcription would support upregulation of tRNAs which may overcome tRNA sequestration by CMT-mutant aaRS, as shown in animal models where increased tRNA levels reduce neuropathy (Zuko et al., 2021). Identifying the minimal stimulus necessary to restore protein synthesis will open new avenues for treating this neuropathy.

Developing neurons are remarkably resistant to pathological stressors that manifest in adulthood, suggesting a decline in axon outgrowth inversely correlates with resilience against proteotoxic threats. Protein synthesis tapered off in YARS-G41R neurons 4 d after replating, consistent with the gradual reduction of regenerative markers in replated neurons (Frey et al., 2015) and slower axon growth. Boosting regenerative capacity to repair the nervous system has been a long-term aspiration in neuroscience. Our study reinforces the value of this ambitious goal as a therapeutic option for CMT and other neurological disorders.

## Synthesis

Reviewing Editor: Laura Volpicelli-Daley, The University of Alabama at Birmingham Decisions are customarily a result of the Reviewing Editor and the peer reviewers coming together and discussing their recommendations until a consensus is reached. When revisions are invited, a fact-based synthesis statement explaining their decision and outlining what is needed to prepare a revision will be listed below. The following reviewer(s) agreed to reveal their identity: Robert Burgess. Note: If this manuscript was transferred from JNeurosci and a decision was made to accept the manuscript without peer review, a synthesis may not be available.

The detailed comments of both reviewers were in agreement. Overall, both authors felt the study advances the field by providing a relevant CMT model for studying the impact of tRNA synthase mutants in dorsal root ganglion cells. The role of mTOR in reduced protein synthesis is important as well as showing the YARS-D81I mutant reduces protein synthesis. Both reviewers requested further exploration of the integrated stress response. In addition, concerns were raised about the number of replicates and whether some experiments were powered to detect significant differences. In addition, clarification of the statistical analyses was also requested. Other important comments were made to increase the strength of the findings such as including data from supplementary figure 1 showing that all CMT-YARS mutants behave similarly into figure 1.

Specific comments by the reviewers that could help improve the manuscript:

Reviewer 1:

The paper by Koenig et al. describes a cellular model of dominant intermediate Charcot-Marie-Tooth disease type C caused by dominant mutations in YARS1, encoding tyrosyl tRNA synthetase. The authors show that lenti-viral transduction of disease-associated YARS1 mutations in primary dorsal root ganglion neurons leads to axon degeneration and caspase-dependent cell death. This is mitigated by inhibitors of the integrated stress respons. The authors also show that protein synthesis is decreased using the Time Stamp (TS) system. The authors also find that axon regrowth following replating is not impaired by transduction with mutant YARS1and that protein synthesis is restored at short time points after replating. Inhibition of the mTor pathway with Torin prevents this restoration of protein synthesis.

This is an interesting and efficient model system for this disease and DRG neurons are a relevant cell type for studies of CMT. The system relies on overexpression, but given the proposed gain-of-function mechanisms of tRNA synthetase mutations in CMT and previous work in cell lines and in Drosophila, this is not a major weakness of the study design and the experiments are appropriately controlled.

The characterization of the cell death as caspase-dependent apoptosis is an important contribution.

I have two primary concerns with this paper.

First, the experiments have a generally low number of replicates and some have fairly high variability. This is particularly a concern for figure 7 where the effects of actinomycin D are not significant whereas the effects of Torin are. However, the overall pattern of effects is very similar for both and the Torin results may be skewed by a few high values in the controls (vector and WT YARS1). These results should be reproduced to determine if either or both are truly significant.

Second, the authors explore changes in protein synthesis after replating and conclude that the effects observed are through mTor. However, in figure 1 the authors show GCN2 and the integrated stress response are also activated. As phosphorylation of eIF2a also decreases protein synthesis, the authors should also examine whether the ISR is also re-set by replating by western blotting or staining for markers such as P-eIF2a or ATF4.

Determining whether changes in translation and activation of the integrated stress response are separable in these diseases is an important question.

Minor points:

The title probably overstates the findings around mTor, which is a small part of the last figure tested using only one approach.

In the introduction, line 101-102, the mouse models of Yars1 should reference Hines et al., J. Anat.

In the introduction, line 108-109, Spaulding et al., Science showed that the ISR is activated in both Gars1 and Yars1 mouse models. It also showed that protein synthesis is decreased in motor neurons in Gars1 mutant mice, which is relevant here, but the efficacy of ISR inhibition was only shown in Gars1 mice, not Yars1.

In figure 1, it is worth noting in the interpretation that the efficacy of GCN2iB suggests the ISR in the DRG system is also being activated through GCN2.

Please indicate the units (Y axes) for axon regrowth in figure 4 C, D, F, G, I.

Line 367, regarding figure 5, please note "at 12 hours".

Reviewer 2:

In this manuscript, the authors use primary sensory neuron cultures prepared from mouse embryonic dorsal root ganglia and transduce them with different YARS-CMT variants to develop models that allow precise manipulations of molecular pathways to study CMT pathogenesis. The authors report that expression YARS-CMT variants leads to axon degeneration and sensory neuron loss by activation of caspase-dependent apoptosis. Apoptotic cell death has not been observed before in Yeast, Drosophila, or mouse models of CMT, where loss of sensory neuron cell bodies is not observed with progression of the phenotypes. Although the model does not fully recapitulate the phenotypes observed in vivo, it is a powerful model to dissect molecular mechanisms of disease.

A very interesting and relevant aspect of the paper is that the data could suggest the involvement of mTOR signaling in early axonal development. The authors speculate that mTOR signaling could prevent protein synthesis defects during development and allow normal axonal growth. The authors suggest that activation of mTOR signaling could explain why protein synthesis is not reduced after re-plating the cells, in contrast to the protein synthesis defect observed after transduction with YARS-CMT variants. This is an innovative idea that could explain why CMT neuropathy presents as an adult-onset neurodegeneration. Additional information supporting and characterizing the involvement of mTOR signaling could enrich the paper and strengthen this novel hypothesis.

Major comments:

1. The authors report that the percentage of cell death is decreased in the cells transduced with CMT YARS G41R by the addition of ISRIB and GCN2iB, suggesting that the activation of the integrated stress response (ISR) promotes neurodegeneration (Fig 1F). However, the authors do not provide direct evidence for the activation of the ISR in their model system. The authors could use stored cell pellet material of cells transduced with CMT YARS G41R from the previous experiments to perform qPCRs in targets of ATF4, or to perform western blots to evaluate p-eIF2a levels. This would strengthen the claim that the ISR is activated in this model.

2. The authors suggest that mTOR signaling is required to restore normal protein synthesis levels in CMT YARS G41R after re-plating based on the effect of Torin-1 in the levels of protein synthesis (Fig 7C). For a mechanistic understanding of how mTOR signaling might prevent protein synthesis defects induced by the CMT-YARS variants after replating, it is important to evaluate the effect of transduction with CMT YARS G41R variant on mTOR signaling before and after replating. This could be tested by assessing if there is a (CMT-YARS G41R variant-specific?) increase in the levels of p-S6 after re-plating.

3. Fig 1A: to evaluate the efficiency of the lentiviral vector transduction, it would be important to determine the percentage of primary sensory neurons in transduced by the lentiviral vector, as well as the expression levels of WT and CMT-mutant YARS proteins, to make sure that they are comparable to each other.

4. Fig 6B: in this western blot, the amount of protein loaded appears to be much lower for the 24 hpr samples as compared to 'pre rep.' samples, which makes the interpretation of the results challenging. Would suggest repeating this western blot with loading of equal amounts of total protein.

5. Fig 6C: how do the authors explain the appearance of an extra YARS band around 50 kDa upon YARS mScar OE?

6. Fig 7C: while TS fluorescence appears clearly reduced in G41R-YARS expressing neurons, this difference is not statistically significant - if this effect is real, increasing the number of replicates should render the difference statistically significant.

7. The data shown in Suppl Fig 1 are very nice and convincing. Fig S1A,B are important because it shows that all CMT-YARS mutants behave similarly. Fig S1C shows that the expression levels of WT and CMT-mutant YARS comparable, which is of key importance (see my previous point). This reviewer would therefore suggest incorporating these data in Figure 1, and in case space would not allow, to split Fig 1 in two main figures.

Minor comments:

1. There are instances where GCN2 phosphorylation can modulate mTOR signaling, which contributes to the overall changes in protein translation. To better understand the involvement of mTOR signaling in their model, it is relevant to test if there are differences in the levels of p-S6 after transduction with CMT YARS G41R at DIV 5. In case there are changes, can those be modulated by inhibiting the activation of the ISR?

These data are relevant to understand whether the changes in protein synthesis observed in the following figures are the exclusive result of ISR activation, or whether modulation of mTOR signaling also contributes to those changes.

2. The authors discuss that "upregulation of axon outgrowth pathways during PNS development insulates these neurons against CMT-YARS and the switch from development to maintenance diminishes these safeguards". To test this idea, a very interesting experiment is to assess if modulation of mTOR signaling can prevent axon degeneration or neuronal cell death. Could overactivation of mTOR signaling by downregulation of TSC1 and TSC2 rescue the protein synthesis defect?

3. Fig 1B+D: what is the pink staining? Please include this info in the figure legend.

4. Fig 3A: the schematic should be adapted to indicate that axonal death was evaluated at DIV5, and cell death at DIV5 and DIV7 in this experiment.

5. The title of figure 3 should be adapted to: 'Reduced protein synthesis precedes axon degeneration in neurons expressing G41R-YARS', because panel F indicates that reduced protein synthesis coincides with caspase activation at DIV5.

6. In Fig. 1C. the authors present a western blot showing that Bcl-xL expression does not affect CMT YARS protein levels in DRGs. In the representative image it looks like the CMT YARS G41R variant has higher level of expression with the Bcl-xL treatment. There are no replicates shown, and there is no quantification of these results. It is important to quantify this data, since an increase in the protein levels of CMT YARS G41R can also explain why the phenotype of axon degeneration seems stronger in this variant compared to CMT YARS D81I and CMT YARS del153.

6. line 118: '...preceded the onset of axon degeneration and caspase activation.' Based on the data shown in Figure 3F, this should be rephrased to: '...preceded the onset of axon degeneration and coincided with caspase activation.'

7. Suggestions for textual changes:

-line 101: '..and mouse models' should be deleted, because there is only a single DI-CMTC mouse model which harbors the E196K mutation.

-line 108: '...ISR is hyperactivated in CMT-YARS mouse models and ISR inhibition alleviates neuropathy phenotypes.' Should be rephrased to: '...ISR is hyperactivated in a CMT-YARS mouse model and ISR inhibition alleviates neuropathy phenotypes in CMT-GARS mouse models.' Because this is what is shown in Spaulding et al, 2021.

-line 242: would suggest to rephrase to 'CMT mutations in tyrosyl-tRNA synthetase (YARS) provoke axon degeneration and sensory neuron death'.

-line 247: references to Spaulding et al, 2021; Hines et al, 2022; and Rhymes et al, 2024 should be omitted here, because the DI-CMTC mouse model contains a E196K mutation in the endogenous mouse Yars gene and is therefore not expressing YARS-CMT variants in a wildtype background.

-line 263: '...expressing all YARS variants...' would suggest to rephrase to: '...expressing any of the YARS variants...' to avoid confusion.

-line 277: would suggest to replace 'constructs' by 'expression'.

-line 279: Spaulding et al, 2021 also reports that ISR activation in Yars-E196K mice, so this should be mentioned here (not only CMT-GARS).

-line 442-443: E196K was also shown to retain enzymatic activity in vivo (Storkebaum et al, 2009), so it would be good to mention this here and include this reference.

-line 444: please rephrase, because protein synthesis defects coincided with caspase activation as shown in Fig 3F.

-line 460: 'receptor' => 'receptors'

-line 463: '...upregulation of tRNAs which sequester CMT-aaRS and reduce neuropathy in animal models': would suggest to rephrase to: '...upregulation of tRNAs which may overcome tRNA sequestration by CMT-mutant aaRS, as shown in animal models where increased tRNA levels reduce neuropathy'.

-line 468: '...a decline in axon outgrowth...'

-line 622: 'on the right' can be deleted.

-line 647: 'as a ratio of 1hr fluorescence' - it is unclear what is meant by this. Please adapt to 'TS fluorescence was measured once per hour during a 12 hour time frame', or the like.

-line 691: 'A slight reduction in axon area was noted at 84 hpr in G41R-YARS neurons' - please add: 'but this difference did not reach statistical significance'.

-line 721: please remove 'pre-'

Finally, some information is missing to properly understand and interpret the figures:

1. In the text the authors mention that "the expression of WT-YARS did not differ from the vector condition, indicating that elevated levels of tyrosyl-tRNA synthetase did not underlie the observed axon degeneration (Fig. 1A)". This phrasing is confusing, because Fig 1A does not show data on YARS expression levels. Would suggest to rephrase to: "as compared to vector condition, the expression of WT-YARS did not induced axon degeneration, indicating....".

2. There is no explanation of the units of the TS Fluorescent axes. It should be clearly stated that this represents fold change to the 1h time point.

3. In line 388 the authors refer to the Figure 6D. However, that figure does not contain a panel D. It only goes up to panel C.
