# Supplementary figures and images for: Replating Induces mTOR-Dependent Rescue of Protein Synthesis in Charcot–Marie–Tooth Diseased Neurons
Source: eNeuro. 2026 Mar 27;13(3):ENEURO.0337-25.2026. doi: 10.1523/ENEURO.0337-25.2026 (PMC13064590; doi:10.1523/ENEURO.0337-25.2026)

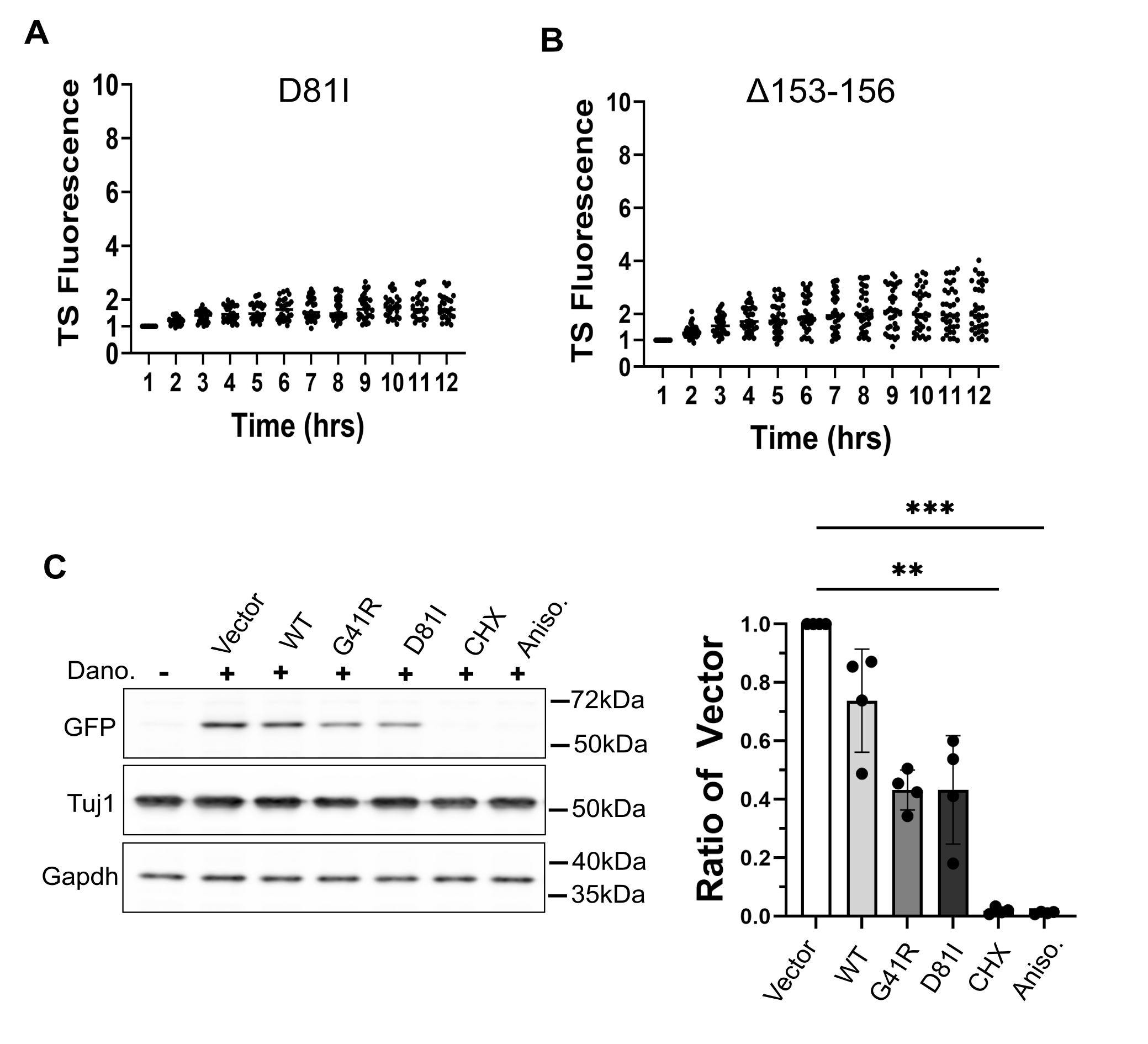

Supplement: Figure 3-1 — Reduced protein synthesis in sensory neurons expressing CMT-YARS. (A) & (B) TS fluorescence from individual cells after danoprevir addition expressing D81I-YARS (28 cells from three independent experiments) or Δ153-156YARS (36 cells from 3 independent experiments). (C) Western blot of DRG extracts four hours after danoprevir addition. TS was detected with an antibody to GFP = with quantification on the right (N = 4). Error bars represent +/-1 SD. We performed a Kruskal-Wallis analysis with Dunn’s post-hoc test to assess significance where ** p˂0.01, and ***p˂0.005. Download Figure 3-1, TIF file. [file eneuro-13-ENEURO.0337-25.2026-s001.tif]

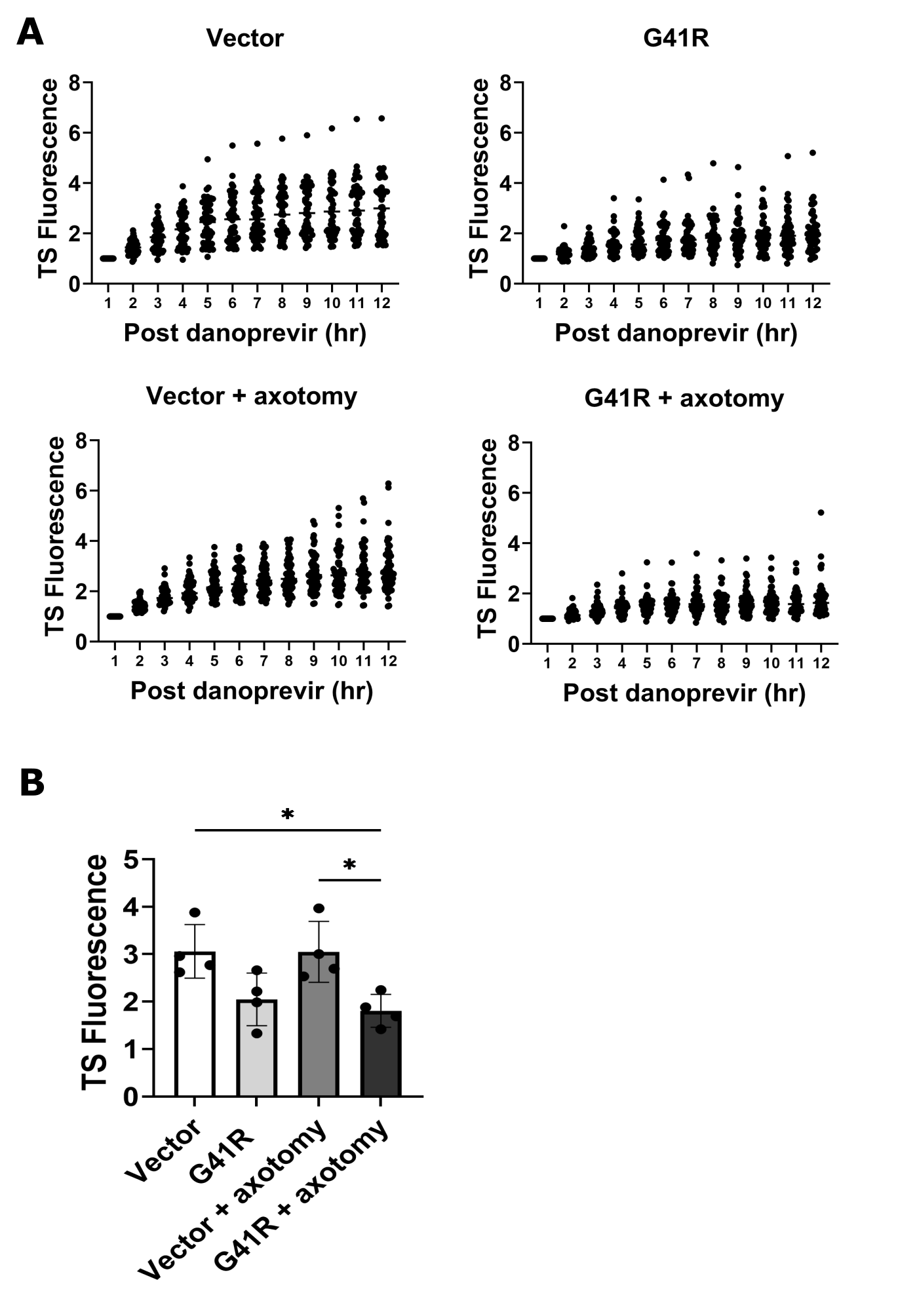

Supplement: Figure 6-1 — Axotomy is not sufficient to restore protein synthesis in YARS-G41R sensory neurons. (A) DRG sensory neurons seeded in a spot culture were transduced on DIV2 with lentivirus expressing an empty vector or YARS-G41R, Bcl-xL to prevent caspase activation, and TimeSTAMP. On DIV8 a razor was used to sever axons around the spot culture. One hour later, danoprevir was added and TimeSTAMP visualized over the next twelve hours. Data points from individual cells are shown for each condition (from at least 40 cells per condition in four independent replicates) with the twelve-hour time point (B). Error bars represent +/-1 SD. For statistical tests, one-way ANOVA was performed with post-hoc t-tests where *p˂0.05. Download Figure 6-1, TIF file. [file eneuro-13-ENEURO.0337-25.2026-s002.tif]

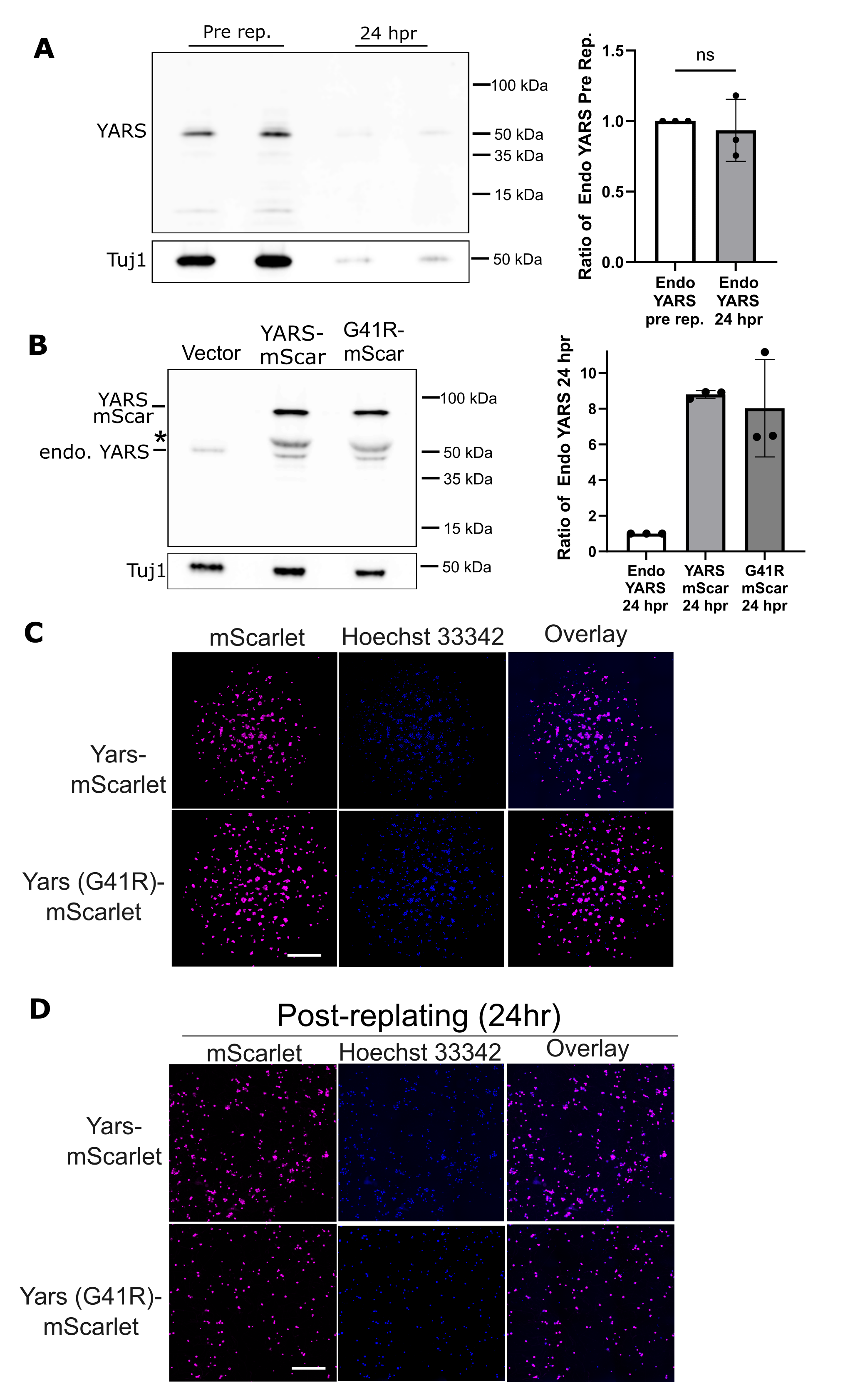

Supplement: Figure 7-1 — YARS-mScarlet expression in DRGs. (A) We compared the ratio of endogenous YARS protein in DRGs pre-replating (pre-rep) to twenty-four hours post replating (hpr). Two replicates are shown in the representative western blot with quantification. (B) We compared endogenous YARS protein to overexpressed, mScarlet-tagged YARS protein after replating by western immunoblotting. The ratio of YARS (G41R)-mScarlet to endogenous YARS was still elevated after replating at a time point when protein synthesis defects were reversed. Asterisk identifies a YARS-positive band below mScarlet-tagged proteins that are likely proteolysis events. (C) Example images of high lentiviral transduction rate in DRG sensory neurons expressing wildtype YARS-mScarlet or YARS (G41R)-mScarlet. Images were collected with an automated microscope as a montage then stitched together to provide a population-wide view of mScarlet expression with Hoechst 33342 labeling nuclei. (D) Stitched images of replated neurons expressing wildtype YARS-mScarlet or YARS (G41R)-mScarlet to demonstrate expression in a majority of cells after replating when protein synthesis defects are reversed. Error bars represent +/-1 SD. Scale bar = 200 µm. Download Figure 7-1, TIF file. [file eneuro-13-ENEURO.0337-25.2026-s003.tif]
